# Analysis of Intellectual Property Rights Reforms Among Health Care Professionals

**DOI:** 10.12688/f1000research.173238.1

**Published:** 2025-12-26

**Authors:** Vinay Kumar Gupta, Atrey J. Pai Khot, Gaurav Mishra, Sumit Kumar, Seema Malhotra, Khusboo Arif, Sonal Bhatia, Aman Rajput, Sultan A. Almalki, Inderjit M. Gowdar

**Affiliations:** 1 Department of Public Health Dentistry, King George's Medical University Faculty of Dental Sciences, Lucknow, Uttar Pradesh, 226003, India; 2 Department of Public Health Dentistry, Goa Dental College and Hospital, Bambolim, Goa, 403202, India; 3 Community Health Centre, Chinhat, Lucknow-226003, Uttar Pradesh, India; 4 Department of Preventive Dental Sciences, College of Dentistry, Prince Sattam bin Abdulaziz University, Al Kharj, Riyadh Province, 11942, Saudi Arabia

**Keywords:** copyrights, healthcare, health professional, patent, intellectual property, trademark

## Abstract

**Introduction:**

The rapidly evolving nature of technology and globalization in recent times makes it imperative to remain at the forefront of innovation and creativity in order to effectuate changes in health practices.

**Objective:**

To assess and compare knowledge, attitudes, and practices regarding Intellectual Property Rights (IPR) reforms among various academic ranks and grades of healthcare professionals in North India.

**Methods:**

A self-designed validated questionnaire comprising 24 close-ended questions was administered to healthcare professionals working in various settings through online links. This cross-sectional study was conducted from August 2023 to September 2023. Prior to this study, a pilot study of 20 healthcare professionals was conducted with a sample size of 747. The reliability of the questionnaire was assessed using Cronbach’s α (0.84), and validity was assessed using the content validity ratio (0.76). The data were analyzed using IBM SPSS Statistics for Windows, version 28.0. Armonk, NY: IBM Corp.

**Results:**

The average age of the participants was 36.31 ± 9.95 years, and their average experience was about 8.53 ± 5.70. The mean overall knowledge score for healthcare professionals was 9.65 ± 1.26 (faculty), and 7.73 ± 1.84 (Residents). The majority of participants 289 (38.7%) agreed that obtaining a patent was tedious process.

**Conclusion:**

Healthcare professionals have scarce awareness of intellectual property rights and their reforms in research. The Ayurveda specialty participants and government institution participants were better aware of IPR reforms, but implementation was challenging for them. Hence, enforcing a robust IPR regime and its appropriate implementation is of utmost importance for an hour.

## Introduction

The rapidly evolving nature of technology and globalization in recent times, it is imperative to remain at the forefront of innovation and creativity in order to effectuate changes in health practices. ^1^ Intellectual Property Rights (IPR) can be defined as “the rights given to people over the creation of their minds”. ^2^ It is a generic legal word, encompassing protection of the legal rights to expressions, and ideas inventors. ^1, 2^

The International Association for the Advancement of Teaching and Research in Intellectual Property (ATRIP), a non-governmental organization was established by the International Bureau of WIPO in 1981. ^3^ Furthermore, it established the WIPO World Wide academy (WWA) to functions as an autonomous educational setting for training and research in Intellectual Property (IP). It offers professional training, policy training, and remote learning education to raise IP awareness. ^3^ As per the IP index, India ranked 29th out of 30 nations worldwide according to report by the US Chamber of Commerce which is extremely worrying state for the country and policy makers. ^4^

The Ministry of Electronics & Information Technology Deity-IPR Cell in developing nations such as India is making significant efforts to enhance infrastructure by generating awareness and facilitating assistance in promoting IPR. The Government of India has made substantial initiatives to create a conducive atmosphere for the establishment, preservation, and administration of IPR. The Union Cabinet adopted the much-awaited “National Intellectual Property Rights (IPR) Policy” on May 12, 2016, to establish a future course for IP administration in India. ^3, 4^ These changes have changed periodically, with the most recent revision occurring in 2019. The Patents Act of 1970 governs the granting of patents to India. ^2, 4^

IP is typically classified into “Industrial Property” and “Copyright.” Industrial property encompasses industrial designs, trademarks, trade secrets, inventions, and geographic indications, while copyright comprises literary and artistic works. ^2, 5^ Any idea or creation that is useful, novel, and non-obvious is patentable; therefore, IPR plays a major role in the healthcare system. The revolution and advances in technology have resulted in the transformation of the intellectual sector and skill proficiency, both of which are rapidly materializing and evolving. ^5^ In this age of social media, the protection of intellectual property rights presents several obstacles. Some of these concerns include trade secrets and infringements. ^3– 5^ This research is exploratory, as it attempts to investigate a problem that is not clearly defined or about which we have less knowledge. ^6^

Advancements in traditional knowledge, science and technology, arts and culture, and biological resources are facilitated by intellectual property. ^7^ It fosters innovation by providing creators and inventors with recognition and financial benefits. Conversely, a lack of understanding of IP rights and its poor application may impede a country’s advancements in technology, economics, and society. ^7, 8^ Therefore, it is crucial to enforce a strong intellectual property rights framework, disseminate IPR knowledge, and ensure that it is implemented appropriately. There is a dearth of research assessing literacy about IPR in healthcare service providers in India. Thus, this study aimed to assess Intellectual Property Rights reforms in India among healthcare professionals in Lucknow Uttar Pradesh.

## Materials and methods

### Study design and study setting

This observational, cross-sectional study was conducted over a period of two months from August 2023 to September 2023 in compliance with the Strengthening the Reporting of Observational Studies in Epidemiology (STROBE) guidelines.

### Study participants and sampling

Study Participants comprised 747 healthcare professionals designated as residents and faculty belonging to widespread government and private healthcare specialties, including Medical, Dental, Pharmacy, Nursing, Ayurveda, and Homeopathy in Lucknow, Uttar Pradesh. A pilot study was conducted on a sample of 20 healthcare professionals from a dental specialty to pretest the questionnaire to detect any problems with design, such as ambiguity of words, feasibility, and inability to understand the questions. The questionnaire was modified on the basis of their feedback. The main study did not include the findings of the pilot trial. The GPower program (G* Power Version 3.1.9.4 statistical software) was used to compute the sample size for the study at a power of 0.95, an alpha error of 0.05, and data uncured from the pilot study, with a sample size of 747. Eligible and eager participants with valid signed Google accounts were provided with a link to the structured questionnaire, ensuring absolute anonymity and confidentiality. The study included participants who provided informed consent. Undergraduates, internals, and private practitioners of healthcare sciences were excluded from the study.

### Ethical consideration and informed consent

This study was approved by the Institutional Ethics Committee of King George’s Medical University (Ref. no: XXI-PGTSC-ILAP43). This study complied with the Helsinki Declaration of 1975, revised in 2000, and ethical guidelines for human experimentation. Prior to study enrollment, a mandatory section with written informed consent and a short overview of the study was presented to the participants, after which it was permissible to proceed with the study.

### Questionnaire design and validation

The self-designed questionnaire was developed in English using Google Forms (a free web-based survey generator) ( https://docs.google.com/forms/u/0/tgif=d) and transformed into electronic media. A link to the questionnaire and a consent form were created and disseminated to healthcare professionals through email as a platform. The questionnaire consisted of two sections with 24 close-ended questions. The first section encompassed the collection of demographic information from the participants. This was followed by the second section consisting of 14 questions about knowledge, 6 questions related to attitude, and 4 questions pertaining to practices regarding IPR in India. The face and content validity of the questionnaire were assessed by an expert committee of eight subject matter experts for readability, clarity, and comprehensiveness of the questions. Test-retest reliability was measured using Kappa statistics to evaluate the reliability of the questionnaire. Cohen’s kappa coefficient was 0.88, indicating a high level of agreement. Additionally, the Cronbach’s alpha coefficient was calculated, yielding a value of 0.84, which demonstrates good internal consistency. The validity of the questionnaire was determined by means of face off validity (0.82%) and content validity ratio (0.76).

### Data collection, procedure, and response grading

Data were collected using a structured online questionnaire distributed exclusively by healthcare professionals, encompassing the inclusion criteria. No enticements or hints were offered to participants during the completion of the questionnaire. The questionnaire completion time was estimated to be approximately 10-15 minutes per individual. Discretion of the acquired information was ensured during the study. The criteria for the study were formulated to make the study simple and clear. An introductory message described the study’s aim and the participants’ voluntary involvement. Following the original invitation, a one-week reminder was delivered. Participants were requested to return the form within two to three weeks after obtaining it; forms returned after the collection period had expired were removed from the analysis. Moreover, a remark on exercising discretion was included in the participants’ questionnaire.

The grading system employed in this study is based on quartile derivatives. The knowledge, attitude, and practice scores were computed by assigning one point for each accurate or positive response, and zero points for each incorrect or negative response. The final scores were given as percentages after summing each participant’s points and calculating the percentages to assess the overall knowledge of the participants. The Knowledge level was categorized as follows: high (>66%), medium (33%–65%), and low (<33%). Similarly, responses to questions related to attitude were scored as 1 for strongly disagree, 2: disagree, 3: neutral, 4: agree, and 5: strongly agree, followed by summing up total score to categorize into positive (>50%) and negative (<50%). ^9^

### Data processing and analysis

The recorded data were entered into Microsoft Excel (2019) and analyzed using IBM Corp. 2012, IBM SPSS ^®^ Statistics for Windows, Version 21.0. Armonk, NY: IBM Corp. The chi-squared test was used to determine the association between variables. The Kruskal-Wallis test was employed to determine the differences in knowledge, attitude, and practice among participants. Multivariate analyses were performed for the various study variables. Statistical significance was set at *p *≤ 0.05.

## Results

### Sociodemographic characteristics of participants

A total of 747 responses were received from healthcare professionals, with an average age of 36.31 **±** 9.95 years. majority of the participants were belonged to dental specialty (37.6%), with an average year of experience of 8.53 ± 5.70. More than half of the participants were designated residents (57.8) employed in government institutions (50.6%) ( Table 1).

**Table 1.** Characteristics of the study participants (N = 747).

Variable		*n* (%)
Sociodemographic characteristics		
Gender	Male	511(68.4)
	Female	236 (31.6)
Age	20-30	251 (33.6)
	31-40	200 (26.8)
	41-50	187 (25)
	51-60	109 (14.6)
Specialty	Medical	106 (14.2)
	Dental	281 (37.6)
	Pharmacy	142 (19.0)
	Nursing	68 (9.1)
	Ayurveda	111 (14.9)
	Homeopathy	39 (5.2)
Type of Institution	Government	378 (50.6)
	Private	369 (49.4)
Designation	Residents	431(57.8)
	Faculty	315 (42.2)
Years of service	<5 years	282 (37.7)
	6 to 10	218 (29.2)
	11 to 15	149 (19.9)
	>16	98 (13.1)
Total		747

All values are expressed as the frequency with percentages (in parentheses).

### Knowledge among participants regarding IPR reforms in India

**Figure 1.**
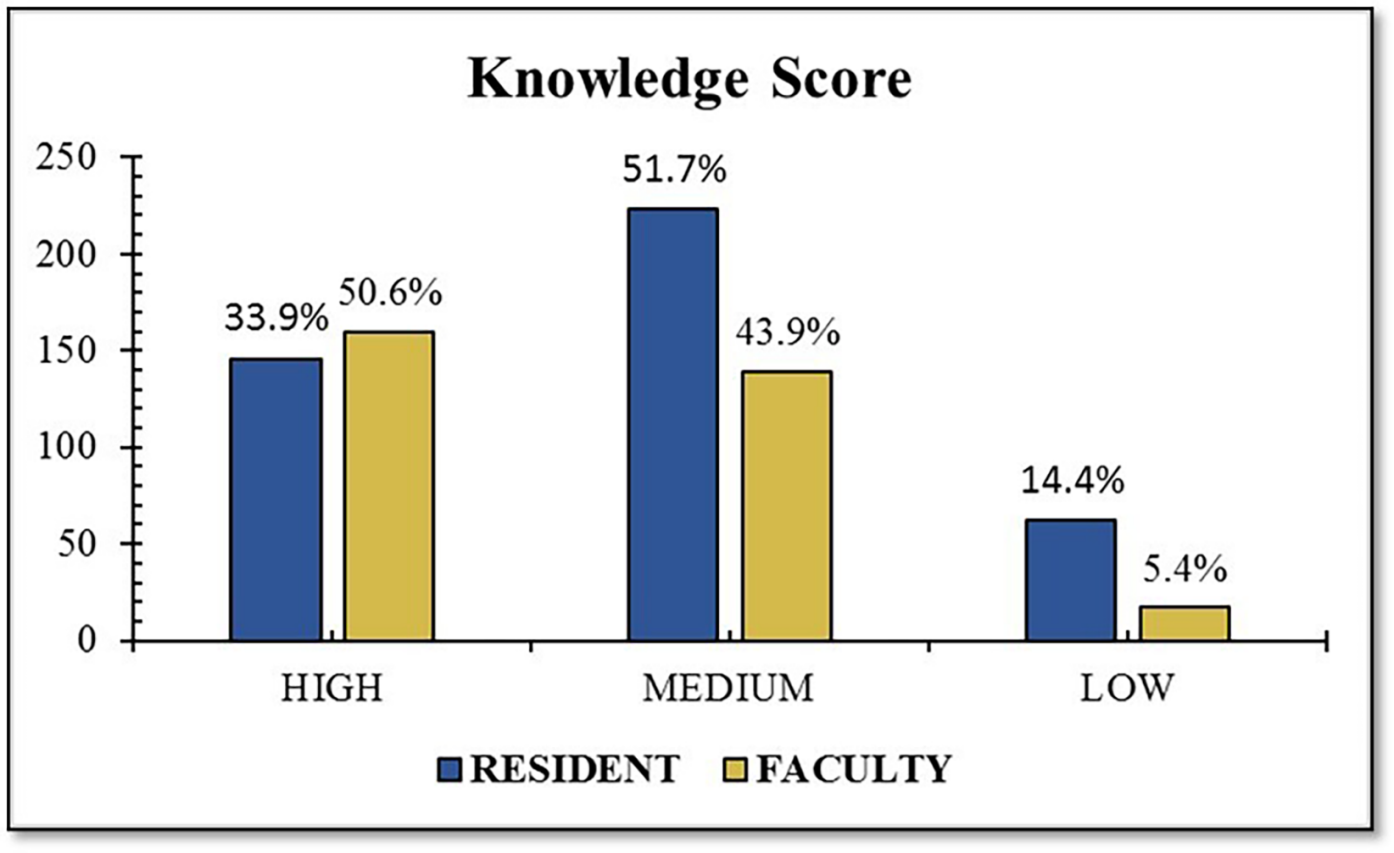
Knowledge score of healthcare professionals towards IPR reforms in India.

Table 2 shows the participants’ responses based on their knowledge of IPR reforms in India. The majority of the faculty participants (92.4%) were aware of the term IPR and institutional policy as compared to residents (65%), which was statistically significant ( *p* < 0.001). The Information about the headquarters and longevity of patent rights was perceived significantly higher by residents (49.7%) in comparison to faculty (25%) with *p* < 0.001). However, knowledge of copyright infringement was statistically insignificant among the participants ( *p* = 0.092). The Mann-Whitney U test highlighted that knowledge scores were higher among females in the medical (10.95), nursing (7.70), and Ayurveda (10.11) specialties. Males were found to be more knowledgeable in the specialties of dental (8.50), pharmacy (8.75), and homeopathy (4.82). There was a statistically significant difference among the specialties with the highest knowledge score in the Ayurveda field in the age group of 31-40 ( *p *< 0.001). Healthcare professionals in government institutions in all specialties had higher knowledge, with an average score 9.54 ± 2.66 score than private institution ( *p *< 0.001). The knowledge score increased with years of service among healthcare specialties according to the Kruskal Wallis test ( *p *< 0.001) ( Table 3). The knowledge score for the designation is shown in Figure 1.

**Table 2.** Responses of participants based on knowledge.

Knowledge based questions	Response	*Resident n* (%) = 431	*Faculty n* (%) = 316	*P-Value *
What does the term IPR mean	Indian Patent Registry ^‡^	141 (32.7)	24 (7.6)	**≤0.001** **
	Intellectual Property Rights ^¶^	280 (65)	292 (92.4)	
	Indian Property Rights ^‡^	0	0	
	None of the above ^‡^	10 (2.3)	0	
IPR in India covers	Patents ^‡^	105 (24.4)	10 (3.2)	**≤0.001** **
	Copyrights ^‡^	5 (1.2)	17 (5.4)	
	Trademarks ^‡^	5 (1.2)	0	
	All of the above ^¶^	306 (71)	289 (91.5)	
	None of the above ^‡^	10 (2.3)	0	
IPR protects the use of information and ideas that are of	Ethical Value ^‡^	97 (22.5)	125 (39.6)	**≤0.001** **
	Monetary Value ^‡^	70 (16.2)	30 (9.5)	
	Commercial Value ^¶^	190 (44.1)	145 (45.9)	
	Don’t Know ^‡^	74 (17.2)	16 (5.1)	
Headquarters of Indian Patent Office	Mumbai ^‡^	89 (20.6)	119 (37.7)	**≤0.001** **
	Chennai ^‡^	42 (9.7)	10 (3.2)	
	Kolkata ^¶^	214 (49.7)	79 (25)	
	Don’t Know ^‡^	86 (20)	108 (34.2)	
Patentability criteria includes	Usefulness ^‡^	69 (16)	36 (11.4)	**≤0.001** **
	Novelty ^‡^	69 (16)	57 (18)	
	Non obviousness ^‡^	28 (6.5)	0	
	All the above ^¶^	224 (52)	184 (58.2)	
	None of the above ^‡^	41 (9.5)	39 (12.3)	
Who can apply for patent	True and first inventor ^‡^	103 (23.9)	32 (10.1)	**≤0.001** **
	Assignee ^‡^	62 (14.4)	14 (4.4)	
	Legal representative ^‡^	35 (8.1)	20 (6.3)	
	All of the above ^¶^	200 (46.4)	240 (75.9)	
	None of the above ^‡^	31 (7.2)	10 (3.2)	
Patent right is	Limited period right ^‡^	178 (41.3)	104 (32.9)	**.007** *
	Territorial right ^‡^	59 (13.7)	51 (16.1)	
	Absolute right ^¶^	124 (28.8)	123 (38.9)	
	Don’t know ^‡^	70 (16.2)	38 (12)	
Prior art search includes	Search of Patent literatures ^‡^	0	0	**.001** *
	Search of Non-patent literature ^‡^	172 (39.9)	92 (29.1)	
	Both (1) and (2) ^¶^	212 (49.2)	200 (63.3)	
	None of the above ^‡^	47 (10.9)	24 (7.6)	
Tools for Patent Search	Websites ^‡^	56 (13)	5 (1.6)	**≤0.001** **
	Newsletters ^‡^	5 (1.2)	0	
	Online Free databases ^‡^	33 (7.7)	0	
	All of the above ^¶^	325 (75.4)	289 (91.5)	
	None of the above ^‡^	12 (2.8)	22 (7)	
How long do patents usually last for?	10 years ^‡^	62 (14.4)	75 (23.7)	**≤0.001** **
	20 years ^¶^	263 (61)	137 (43.4)	
	40 years ^‡^	6 (1.4)	26 (8.2)	
	Don’t know ^‡^	100 (23.2)	78 (24.7)	
The first offence for infringement of copyright can be for a maximum of imprisonment for a term of	3 years and a fine of Rs 50,000 ^‡^	143 (33.2)	90 (28.5)	.092
	3 years and a fine of Rs 2,00,000 ^¶^	71 (16.5)	62 (19.6)	
	1 years and a fine of Rs 3,00,000 ^‡^	48 (11.1)	23 (7.3)	
	Don’t know ^‡^	169 (39.2)	141 (44.6)	
What does a trademark protect?	An invention ^‡^	86 (20)	38 (12)	**≤0.001** **
	A work of art ^‡^	26 (6)	17 (5.4)	
	Logos, names and brands ^¶^	242 (56.1)	230 (72.8)	
	Don’t know ^‡^	77 (17.9)	31 (9.8)	
Most common databases used to retrieve patent information	Patent Scope (WIPO) ^¶^	113 (26.2)	90 (28.5)	**.005** *
	Indian Patent office ^‡^	58 (13.5)	42 (13.3)	
	USPTO (United States Patent and Trademark Office) ^‡^	128 (29.7)	107 (33.9)	
	Google ^‡^	50 (11.6)	12 (3.8)	
	Don’t Know ^‡^	82 (19)	65 (20.6)	
Are you aware about your institutional IPR policy and its established cell	Yes ^¶^	237 (55)	223 (70.6)	**≤0.001** **
	No ^‡^	137 (31.8)	79 (25)	
	Don’t know ^‡^	57 (13.2)	14 (4.4)	

All values are expressed as frequencies and percentages (parentheses).

^¶^ Denotes the correct response, and

^‡^ Denotes the wrong response. Statistical tests were performed using the chi-squared test. Level of significance:

* *p* ≤ 0.05, considered statistically significant;

** *p* ≤ 0.001, highly significant association.

**Table 3.** Comparison of demographic characteristics and knowledge scores among the participants.

Knowledge score	n (mean ± SD)							
	Medical	Dental	Pharmacy	Nursing	Ayurveda	Homeopathy	Rank	p-value
Average	106 (8.36 ± 3.19)	281 (8.74 ± 2.68)	142 (8.94 ± 2.78)	68 (6.66 ± 1.45)	111 (9.61 ± 3.08)	39 (4.82 ± 3.78)		
Based on Gender ^α^								
Male	85 (7.72 ± 3.26)	127 (9.03 ± 2.78)	126 (8.96 ± 2.95)	41 (5.98 ± 0.16)	93 (9.52 ± 3.33)	39 (4.82 ± 3.78)	0.959	**0.338**
Female	21 (10.95 ± 0.22)	154 (8.50 ± 2.59)	16 (8.75 ± 0.45)	27 (7.70 ± 1.88)	18 (10.11 ± 1.02)	0		
Based on Age ^β^								
20-30	27 (7.78 ± 3.18)	87 (8.95 ± 2.48)	64 (8.52 ± 3.20)	30 (6.50 ± 1.31)	24 (9.46 ± 2.25)	19 (4.42 ± 3.79)	16.137	**0.001** *
31-40	29 (7.69 ± 3.75)	79 (7.94 ± 2.84)	31 (8.90 ± 2.34)	21 (6.86 ± 1.59)	28 (10.18 ± 3.04)	12 (4.58 ± 3.58)		
41-50	26 (8.27 ± 2.84)	76 (9.20 ± 2.34)	33 (9.27 ± 2.30)	12 (7.08 ± 1.73)	34 (10.06 ± 3.29)	6 (7.50 ± 3.99)		
51-60	24 (9.92 ± 2.38)	39 (9 ± 3.15)	14 (10.14 ± 2.48)	5 (5.80 ± 0.45)	25 (8.52 ± 3.36)	2 (2 ± 0)		
Based on type of institution ^α^								
Government	73 (9.79 ± 1.91)	189 (8.87 ± 2.23)	8 (9 ± 0.53)	24 (7.5 ± 1.84)	67 (9.91 ± 2.95)	17 (8.47 ± 2.96)	5.399	**≤0.001** *
Private	33 (5.18 ± 3.18)	92 (8.47 ± 3.43)	134 (8.93 ± 2.86)	44 (6.20 ± 0.93)	44 (9.16 ± 3.23)	22 (2 ± 0)		
Based on Designation ^α^								
Resident	49 (5.98 ± 3.16)	166 (8.67 ± 2.43)	166 (8.67 ± 2.43)	135 (8.95 ± 2.85)	41 (5.98 ± 0.16)	18 (10.11 ± 1.02)	5.747	**≤0.001** *
Faculty	57 (10.40 ± 1.15)	115 (8.84 ± 3.02)	115 (8.84 ± 3.02)	7 (8.71 ± 0.95)	27 (7.70 ± 1.88)	93 (9.52 ± 3.33)		
Based on years of experience ^β^								
<5 years	24 (6.29 ± 3.63)	72 (7.03 ± 3.12)	110 (8.66 ± 2.63)	48 (6.5 ± 1.13)	16 (9.81 ± 0.83)	12 (7.75 ± 3.49)	4.405	**≤0.001** *
6 to 10	49 (9.12 ± 2.61)	102 (8.75 ± 2.41)	26 (8.66 ± 3.09)	11 (8.27 ± 1.62)	16 (9.31 ± 2.12)	14 (3.86 ± 3.37)		
11 to 15	20 (7.30 ± 3.23)	76 (9.92 ± 2.20)	4 (7.25 ± 1.89)	3 (6.33 ± 2.31)	33 (9.52 ± 3.13)	13 (3.15 ± 3)		
>16	13 (10.92 ± 0.28)	31 (9.77 ± 0.99)	2 (10.5 ± 2.12)	6 (5.17 ± 0.41)	46 (9.72 ± 3.79)	0		

All values are expressed as the mean ± standard deviation (SD) and median with range (in parentheses). The statistical test used was as follows:

^α^ Mann Whitney U test;

^β^ Kruskal Wallis test; Level of significance:

** P ≤ 0.001 was considered highly statistically significant;

* P ≤ 0.05, was considered statistically significant.

### Attitude and practices among participants regarding IPR reforms in India

**Figure 2.**
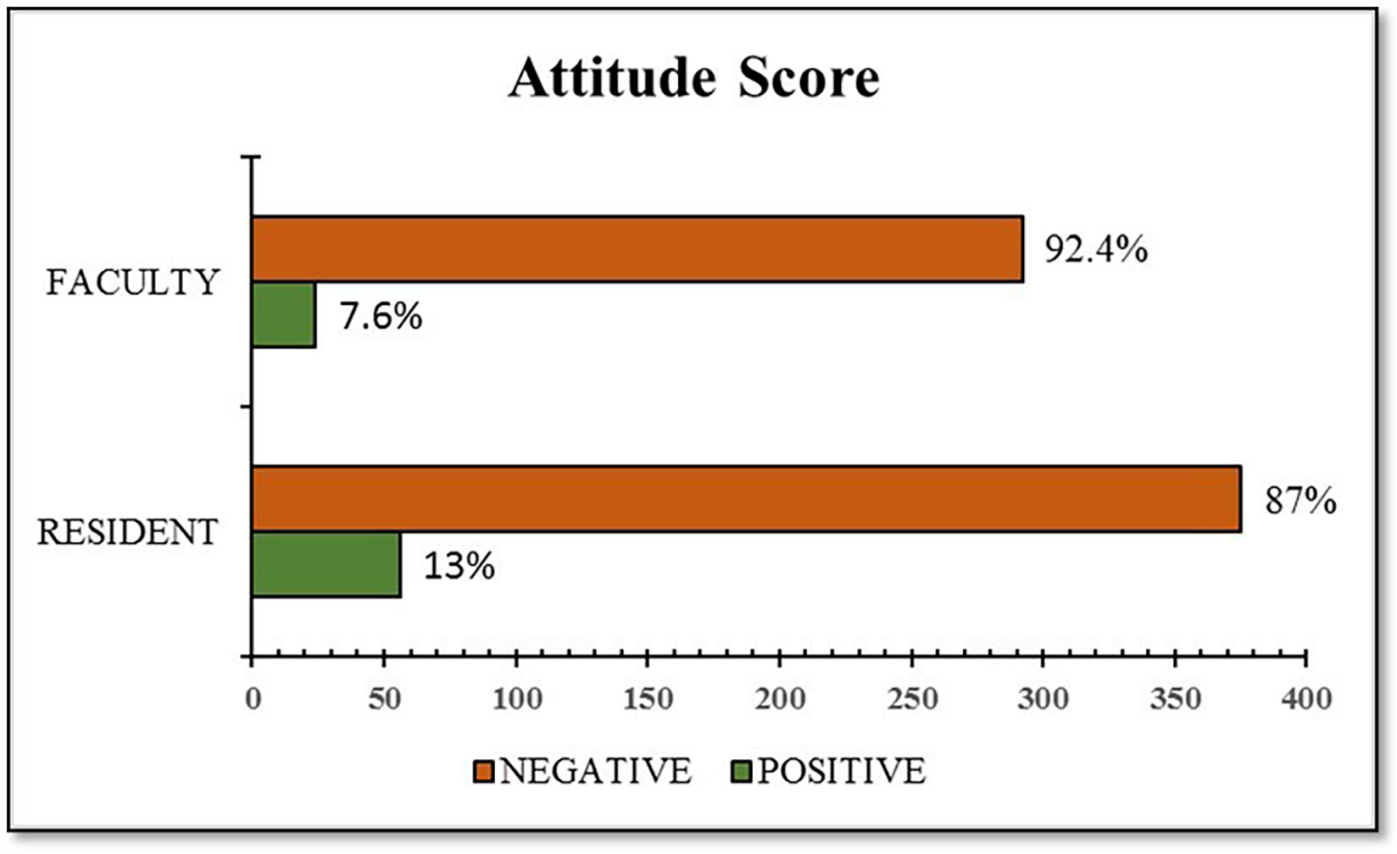
Attitude score of healthcare professionals towards IPR reforms in India.

There was statistically significant difference in the attitude of faculty (36.4%) than residents (28.8%), strongly agreeing that patenting a tedious process than publishing in a scientific journal ( *p* = 0.012). Majority of the participants agreed on trademarks their organization names (88.9%). However, almost half of the faculty (50.9%) and one the residents (34.8%) believed patenting process lengthy and possessed financial burden with statistically significant difference ( *p* < 0.001). More than three-fourths of the participants adapted to the new changes and positive practices of the IPR. There was insignificant difference ( *p* = 0.875) between faculty (86.7%) and residents (86.3%) with regard to the need for sensitization program for IPR ( Table 4). A comparison of attitudes among participants revealed that males had better attitudes than females. Healthcare specialists from pharmacies perceived a positive attitude towards acquiring IPR reforms. The Kruskal Wallis test showed a statistically significant difference in attitudes between government and private institutions ( *p* = 0.031). Moreover, the residents in different specialties had a positive attitude compared to the faculty ( *p* = 0.002). Clinical experience significantly influenced the attitude score, wherein recently graduated healthcare specialists (<5 years) were found to have a positive attitude score, which was statistically significant ( *p < 0.001*) ( Table 5). The attitude score with the designation is shown in Figure 2.

**Table 4.** Responses of participants based on attitude and practices.

Attitude based questions	Response	*Resident n* (%) = 431	*Faculty n* (%) = 316	*p-Value *
Do you think the protection of IPR is through law and registrations?	Strongly Agree	178 (41.3)	246 (77.8)	**≤0.001** **
	Agree	115 (26.7)	32 (10.1)	
	Neutral	86 (20)	15 (4.7)	
	Disagree	0	23 (7.3)	
	Strongly Disagree	52 (12.1)	0	
Do you think your organization name should be protected by a trademark?	Strongly Agree	154 (35.7)	168 (53.2)	**≤0.001** **
	Agree	173 (40.1)	84 (26.6)	
	Neutral	84 (19.5)	54 (17.1)	
	Disagree	0	10 (3.2)	
	Strongly Disagree	20 (4.6)	0	
Do you believe that a patent should be in public domain before it is filed	Strongly Agree	99 (23)	90 (28.5)	**≤0.001** **
	Agree	138 (32)	50 (15.8)	
	Neutral	175 (40.6)	53 (16.8)	
	Disagree	3 (0.7)	52 (16.5)	
	Strongly Disagree	16 (3.7)	71 (22.5)	
Do you believe that obtaining a patent is tedious process than publishing in a scientific journal?	Strongly Agree	124 (28.8)	115 (36.4)	**.012** *
	Agree	162 (37.6)	127 (40.2)	
	Neutral	43 (9.98)	27 (8.54)	
	Disagree	44 (10.2)	27 (8.5)	
	Strongly Disagree	58 (13.46)	20 (6.33)	
Do you believe that the duration required for the patenting process is excessively lengthy?	Strongly Agree	90 (20.9)	104 (32.9)	**≤0.001** **
	Agree	150 (34.8)	144 (45.6)	
	Neutral	97 (22.5)	58 (18.4)	
	Disagree	75 (17.4)	10 (3.2)	
	Strongly Disagree	19 (4.4)	0	
Is it your opinion that obtaining a patent places a financial burden and offers fewer benefits?	Strongly Agree	37 (8.6)	94 (29.7)	**≤0.001** **
	Agree	113 (26.2)	67 (21.2)	
	Neutral	173 (40.1)	96 (30.4)	
	Disagree	75 (17.4)	59 (18.7)	
	Strongly Disagree	33 (7.7)	0	
Practice based questions				
Would you choose copyright to establish IPR over information/education/communication material that you have curated?	Yes	398 (92.3)	262 (82.9)	**≤0.001** **
	No	33 (7.7)	54 (17.1)	
Will you patent a product after publishing an article on it?	Yes	374 (86.8)	177 (56)	**≤0.001** **
	No	57 (13.2)	139 (44)	
Will you follow the principle ‘first-to-file and first-to-invent rules’ in IPR	Yes	398 (92.3)	261 (82.6)	**≤0.001** **
	No	33 (7.7)	55 (17.4)	
Do you seek an impending need for IPR sensitization at your institute?	Yes	372 (86.3)	274 (86.7)	.875
	No	59 (13.7)	42 (13.3)	

All values are expressed as frequencies and percentages (parentheses). Statistical tests were performed using the chi-squared test. Level of significance:

* *p* ≤ 0.05, considered statistically significant;

** *p* ≤ 0.001, highly significant association.

**Table 5.** Comparison of demographic characteristics and attitude scores among the participants.

Variable		Mean ± SD	Z	*p*-Value
Gender ^α^	Male	11.26 ± 3.02	1.630	.103
	Female	10.75 ± 2.93		
Age ^β^	20-30	11.45 ± 2.98	13.060	**.005** *
	31-40	10.76 ± 2.97		
	41-50	10.74 ± 3.04		
	51-60	11.53 ± 2.92		
Specialty ^β^	Medical	11.07 ± 2.65	45.041	**≤0.001** **
	Dental	10.83 ± 2.88		
	Pharmacy	12.47 ± 3.31		
	Nursing	10.91 ± 1.55		
	Ayurveda	9.74 ± 2.91		
	Homeopathy	12.41 ± 3.43		
Type of Institution ^α^	Government	10.58 ± 3.03	4.842	**.031** *
	Private	11.63 ± 2.87		
Designation ^α^	Residents	11.75 ± 2.80	7.420	**.002** *
	Faculty	10.21 ± 3.04		
Years of service ^β^	<5 years	12.23 ± 2.88	6.042	**.000** **
	6 to 10	10.14 ± 2.93		
	11 to 15	10.95 ± 2.53		
	>16	10.22 ± 3.10		

All values are expressed as the mean ± standard deviation (SD) and median with range (in parentheses). The statistical test used was as follows:

^α^ Mann Whitney U test;

^β^ Kruskal Wallis test; Level of significance:

** *P* ≤ 0.001 was considered highly statistically significant;

* *P* ≤ 0.05, was considered statistically significant.

### Relationship between knowledge and attitude with other parameters

Multiple linear regression analysis model showed a statistically significant relationship between knowledge score and specialty (β = 0.222; 95%CI: -0.74–0.69; *p* = 0.004), type of institution (β = –0.798; 95%CI: -1.278–(-0.319); *p* = 0.001), and with designation (β = -0.957; 95%CI: -1.455–(-0.460); *p *< 0.001). The attitude score of the participants showed a similar relationship with gender (β = 0.592; 95%CI: 0.107–1.077; *p* = 0.017), designation (β = 1.692; 95%CI: 1.20–2.183; *p *< 0.001), and years of service (β = 0.276; 95%CI: 0.051–0.501); *p *= 0.016) ( Table 6).

**Table 6.** Results of multiple linear regression on factors associated with knowledge and attitude among participants.

Dependent variable	β	SE	*t*	*P*–Value	95% CI for β	Adjusted R Square
Knowledge score						
Constant	9.661	0.348	27.792	<.001 **	8.98–10.34	0.080
Gender (ref: Female)	-0.251	0.250	-1.002	0.317	-0.741–0.240	
Age (ref: 20-30)	0.143	0.105	1.366	0.172	-0.063–0.350	
Speciality (ref: Medical)	-0.222	0.078	-2.851	0.004 *	-0.374–(-0.69)	
Type of Institution (ref: Government)	-0.798	0.244	-3.267	0.001 *	-1.278–(-0.319)	
Designation (ref: Faculty)	-0.957	0.254	-3.776	<.001 **	-1.455–(-0.460)	
Years of service (ref: 6-10)	0.140	0.116	1.210	0.227	-0.087–0.368	
Attitude score						
Constant	9.145	0.343	26.626	<.001 **	8.471–9.819	0.085
Gender (ref: Female)	0.592	0.247	2.396	0.017 *	0.107–1.077	
Age (ref: 20-30)	0.125	0.104	1.203	0.229	-0.079–0.329	
Speciality (ref: Medical)	-0.069	0.077	-0.893	0.372	-0.219–0.082	
Type of Institution (ref: Government)	0.472	0.241	1.957	0.051	-0.001–0.946	
Designation (ref: Faculty)	1.692	0.251	6.752	<.001 **	1.20–2.183	
Years of service (ref: 6-10)	0.276	0.115	2.406	0.016 *	0.051–0.501	

β: regression coefficient; SE: standard error; CI: confidence interval; the statistical test used: multiple linear regression analysis model; Level of significance:

** P ≤ 0.001 is considered highly statistically significant;

* P ≤ 0.05, considered statistically significant.

## Discussion

Over the last decade, there has been an increase in demand for intellectual property rights, particularly in healthcare research and development. IP Index score 2023 for India is 38.64% and is ranked 42 rank according to the U.S. Chamber of Commerce, which is a valuable instrument for analyzing the strength and efficacy of IP frameworks developed by legislators throughout the globe. ^10^ It offers essential data that can be utilized not only to support arguments but also to inspire policy changes that will propel innovation ahead and design a brighter future. India’s reputation in terms of identifying and enforcing IPRs has been dismal. According to *Bala et al.*, ^7^ India has been trailing in the development of IPR assets such as industrial designs, trademarks, registered patents, etc. ^11^ This research aimed to offer an overview of the knowledge and attitude of residents and faculty concerning IPRs in healthcare specialty, which might serve as a foundation for sensitization programs and workshops on IPRs. Intellectual property is a broad term casing a diversity of legally recognized rights derived from creativity, or otherwise connected to ideas. ^12^ In the present study, majority of the participants, including faculty (92.4%) and residents (65%), were aware of the term IPR, nature of IPR, and its commercial value, in contrast to another study by Deshpande et al., wherein the term IPR was known by less than half of postgraduate students and faculties. ^2^

IPR is required for improved innovation or creative identification, planning, marketing, and rendering, and therefore protection. An invention is patentable, provided it satisfies three criteria: novelty, usefulness, and non-obviousness under the stringent examination and opposition processes outlined in the Indian Patents Act, 1970. ^13^ When this was asked to the participants, more than half of the participants answered positively to fulfil all three criteria. Although India has a variety of laws on books intended to safeguard intellectual property rights, the actual enforcement of these statutes is often lax. A patent right is an absolute right that can be notified by few participants. However, almost three forth (75.9%) of the faculties were aware of who was permitted to file the patent as compared to residents (46.4%). According to Kumar et al., most respondents believed that IPR rights could be traded, acquired, or registered. ^1^ However, according to Ahmed et al., only 27 percent of law students agreed with this assertion. ^3^ This might be because comparatively few faculty and residents belonging to the healthcare profession sought to follow the protocol of the filing process. The large population of residents (66.4%) and faculty (76.6%) agreed that obtaining a patent is tedious process than publishing in a scientific journal, which corresponds to study by Gunjan Kumar et al., wherein 17.7% medical, 24.7% dental, and 25.3% of nursing professionals were unaware that law and registrations hold upper hand in the protection of IPR. ^5^

An inventor should become familiar with what has already been invented (the “prior art”) before starting the patent application process. A substantial number of participants agreed to examine patent and non-patent literature for prior art searches. Information is acquired from primary and secondary sources such as newsletters and related websites. Patent searches are accessible through a number of commercial and free databases. One-third of the participants felt that the United States Patent and Trademark Office was the most common database for retrieving patent information. However, limited number of faculty (13.3%) and residents (13.5%) routinely used Indian Patent Office a search database routinely. The study demonstrated a lack of understanding among participants about copyright infringement, which was similar to the findings in a study by Ahmed et al. ^3^ on law students. The lack of familiarity with copyright regulations in the dental industry may be the reason for this poor response. According to Jajpura et al., copyrights safeguard the expression of ideas in the context of mass communications. ^4^ On the contrary, a greater percentage (56.1% and 72.8%) of residents and faculty, respectively, were familiar with trademark protection for logos, names, and brands. The trademark may consist of words, letters, numbers, drawings, pictures, symbols, and even sounds. ^14^ Sharma et al. stated that it offers absolute right to owner for identifying products or services. Many established commercial organizations and food outlets use trademarks on their products, which may explain why the majority of participants were aware of it. ^14^ Health professionals need to trademark clinic names and logos for uniqueness in their private practice.

Consequently, the results of the study clearly delineated that faculty members possess a far greater understanding of IPRs than residents. This is likely due to the fact that most residents (55.7%) believed that that the duration required for the patenting process is excessively lengthy, which is impractical to finish during their tenure, whereas faculty are encouraged to carry out such activities for their promotional benefits in academics. These results were in line with the findings of Kumar et al. ^5^ who disco1vered that dental surgeons (33.3%) had less knowledge than the remaining three responder categories, which included interns, postgraduates, and faculty members, in favoring the use of IPR. It was also noticed that participants who perceived negative attitudes towards IPR may be in their opinion that obtaining a patent places a financial burden and offers fewer benefits.

The dynamic sphere of innovations is attaining more acceptance in government sectors (9.68 ± 2.82) than in private institution (8.39 ± 2.53), perhaps because of funding and financial support associated with the start-up initiative. It was also found that the Ayurveda branch (9.61 ± 3.08) in healthcare excelled in IPR awareness compared to all other specialties, which could be because of the evaluation of more indigenous medicine innovation and promotion. It was noted that 86.3% of residents and 86.7% of faculty desired to explore an approaching requirement for IPR sensitization at their institution. Therefore, training modules, conventions, conferences, and workshops should be explored to sensitize healthcare workers to various constructs of IPR. Incorporating a section about IPR in the standard course may benefit students by making them aware of the subject from the outset, which will indirectly help shield their novel ideas against infringement and inspire the development of health-related technologies for a better future.

### Limitation and recommendation

The study has a cross-sectional design; data collection is through an online platform, which could introduce bias in the responses obtained from participants, and therefore, the findings should be regarded with caution. Future research should focus on interventions to assess the efficacy of IPR education. Furthermore, probability sampling should be considered to improve the generalizability of the results. IPR must be protected by strengthening the enforcement of existing laws, addressing online infringement, harmonizing laws with international standards, fostering innovation and entrepreneurship, and developing specialized IP courts and tribunals.

## Conclusion

Intellectual property rights are fundamental to progressive societal development and essential for both local and global competitive trade. This study revealed that the Intellectual Property landscape of healthcare professionals in India has increased over the years. It is gradually becoming a vital tool for sustainable development and the cornerstone of current economic strategies. The Ayurveda specialty participants and government institution participants were better aware of IPR reforms, but implementation was challenging for them. Hence, enforcing a robust Intellectual Property Rights (IPR) regime and its appropriate implementation is of utmost importance for an hour. Policymakers should include IPR in the primary educational curriculum and promote IPR registration by encouraging innovators.

## Declaration

Ethics approval statement- Ethical clearance was obtained from Institutional Ethics Commitee of King George’s Medical University (Ref. no: XXI-PGTSC-ILAP43). The study was done in accordance with World Medical Association Declaration of Helsinki.

Informed Consent – A written Informed consent for participation in the study has been obtained from all the participants after giving them the full details of the study.

## Data Availability

All underlying de-identified data generated and analysed in this study are openly available in Figshare, an F1000Research-approved generalist data repository. The dataset includes all relevant cleaned data files and documentation necessary to enable verification and reuse. Data are shared under the terms of the Creative Commons Attribution 4.0 International (CC-BY 4.0) license, permitting unrestricted use, distribution, and reproduction provided the original authors are credited. The dataset can be accessed at the following DOI: https://doi.org/10.6084/m9.figshare.30744239 ^15^
