# Supplementary material for: Analysis of Intellectual Property Rights Reforms Among Health Care Professionals
Source: F1000Res. 2025 Dec 26;14:1453. [Version 1] doi: 10.12688/f1000research.173238.1 (PMC12963827; doi:10.12688/f1000research.173238.1)
Supplement: Peer review history

# Reviewer report: Reviewer response for version 1

**DOI:** 10.5256/f1000research.191034.r445933

**Published:** 2026-03-05

**Authors:** Mamata Hebbal

**Affiliations:** 1 Princess Nourah Bint Abdulrahman University, Riyadh, Saudi Arabia

The manuscript presents an engaging topic and is well-written. This research emphasizes the importance of Intellectual Property Rights (IPR) in promoting innovation in healthcare amidst rapid technological and global advancements.

 It highlights the necessity of assessing IPR literacy among healthcare professionals to enhance implementation and innovation within the sector. The questionnaire utilized in this study is robust, supported by a validity assessment and high reliability.

 However, the data collection procedure needs further clarification regarding the distribution of the online questionnaire.

 For example, did the authors use social media, official email addresses, or seek assistance from professional associations? Additionally, the sampling strategy should be clearly outlined. It is important to acknowledge that self-reported Knowledge, Attitudes, and Practices (KAP) surveys are susceptible to social desirability bias.

 The absence of information regarding the response rate is a critical oversight, as this is essential in online surveys.

 Furthermore, excluding late responses may introduce bias, which needs to be acknowledged. There is also an inconsistency in the font used in the tables compared to the main text; this should be standardized. More details should be provided in Table 6 concerning the interpretation of the "R-squared" value. Lastly, adding additional references is recommended.

Is the work clearly and accurately presented and does it cite the current literature?

Yes

If applicable, is the statistical analysis and its interpretation appropriate?

Yes

Are all the source data underlying the results available to ensure full reproducibility?

Yes

Is the study design appropriate and is the work technically sound?

Yes

Are the conclusions drawn adequately supported by the results?

Yes

Are sufficient details of methods and analysis provided to allow replication by others?

Yes

Reviewer Expertise:

Epidemiology of oral diseases, Prevention of oral Diseases, Helath promotion

I confirm that I have read this submission and believe that I have an appropriate level of expertise to confirm that it is of an acceptable scientific standard.

# Reviewer report: Reviewer response for version 1

**DOI:** 10.5256/f1000research.191034.r445928

**Published:** 2026-01-20

**Authors:** Senthil Murugappan

**Affiliations:** 1 Indira Gandhi Institute of Dental Sciences, Puducherry, Puducherry, India

The study reveals substantial gaps in intellectual property (IP) awareness among healthcare professionals, largely attributable to limited formal training and inadequate exposure to ethical research practices in India. Research engagement is often restricted to postgraduate education and driven by the pursuit of statistical significance rather than ethical rigor or translational value. The use of negatively framed questionnaire items may have introduced response and social desirability bias, highlighting the need for neutral question design when assessing baseline knowledge. Additionally, the unequal gender representation (70% male, 30% female) limits the reliability of gender-based comparisons. Participants’ responses further indicate limited understanding of the importance of patenting innovations at an early stage. Finally, reliance on U.S.-centric IP data reduces global relevance; referencing WIPO country-wise reports would provide a more neutral and internationally appropriate perspective.

Is the work clearly and accurately presented and does it cite the current literature?

Yes

If applicable, is the statistical analysis and its interpretation appropriate?

I cannot comment. A qualified statistician is required.

Are all the source data underlying the results available to ensure full reproducibility?

Yes

Is the study design appropriate and is the work technically sound?

Yes

Are the conclusions drawn adequately supported by the results?

Yes

Are sufficient details of methods and analysis provided to allow replication by others?

Yes

Reviewer Expertise:

Oral health care special needs, and innovation in dentistry

I confirm that I have read this submission and believe that I have an appropriate level of expertise to confirm that it is of an acceptable scientific standard.
